# Robot-assisted surgery for Hirschsprung disease in children: initial single-center experience

**DOI:** 10.3389/fsurg.2026.1797606

**Published:** 2026-04-22

**Authors:** Girolamo Mattioli, Maria Stella Cipriani, Stefano Avanzini, Michela Cing Yu Wong, Valentina Rossi, Maria Grazia Faticato

**Affiliations:** 1 Pediatric Surgery Department, IRCCS, Istituto Giannina Gaslini, Genoa, Italy; 2 DINOGMI, University of Genoa, Genoa, Italy; 3 Pediatric Surgery Department, Azienda Ospedaliero Universitaria Renato Dulbecco, Catanzaro, Italy

**Keywords:** children, congenital megacolon, Hirschsprung disease, learning curve, robotic surgery

## Abstract

**Introduction:**

Robotic surgery (RS) application to Hirschsprung disease (HSCR) is spreading. The aim of this study was to describe our series of children operated with RS for HSCR, focusing on surgical outcomes.

**Methods:**

Case series of 20 children operated for HSCR disease with robotic approach over a 10-year period (October 2015 to July 2025). Preoperative characteristics, intraoperative data, and postoperative outcomes were collected. A comparison with patients who underwent a laparoscopic surgery for HSCR disease was done.

**Results:**

The median age at surgery was 1.3 years (IQR 0.6-4.8), with a mean weight of 16 kg. Seven-teen patients had rectosigmoid HSCR (85%), and three had long-segment HSCR (15%). The median total operative time was 253 minutes (IQR 188-402), while the median console operative time was 50 minutes (IQR 40-95). Postoperatively, five patients developed mild anastomotic stenosis, and one required redo ileoanal anastomosis (5%). Median follow-up was 11 months (IQ range 5–12). At last follow-up, two patients complained mild constipation with soiling episodes (12%); and two had experienced episodes of HAEC (12%). Comparative analysis with laparoscopy showed no significant differences in operative outcomes.

**Conclusion:**

RS is a safe option for the management of HSCR. More multicentre studies are necessary to define clear indications.

## Introduction

Hirschsprung disease (HSCR) is a congenital intestinal motility disorder caused by the absence of enteric ganglion cells in the distal intestine, leading to chronic bowel obstruction. In more than 80% of patients, aganglionosis is confined to the rectosigmoid region. In the remaining cases, the aganglionic segment extends beyond the rectosigmoid colon and may involve the entire colon and even the proximal small intestine. The extent of aganglionosis significantly influences both the prognosis and the management strategy.

Surgical treatment consists of resection of the aganglionic intestinal segment, pull-through of the ganglionic bowel and restoration of intestinal continuity through an anastomosis. Several operative techniques have been described over the years, including the endorectal pull-through (ERPT) procedures performed via abdominal and/or perineal approaches. Currently, no definitive evidence supports the overall superiority of one technique over another in terms of postoperative complications or long-term bowel function (1).

In 2011, robotic surgery (RS) had been introduced for HSCR management (2). Reported applications include the abdominal phase of Swenson and Soave ERPT, Deloyers’ maneuver, innervation mapping, Duhamel pull-through, redo surgery, excision of a redundant rectal pouch, and radical proctocolectomy. According to a recent systematic review RS for HSCR appears feasible and safe in infants—and have even been described in the neonatal period (3)—although it does not demonstrate superiority over laparoscopy in this age group. In older children and redo cases, however, the robotic platform may provide advantages due to enhanced visualization and improved dexterity for deep pelvic dissection (4).

This case series describes the first twenty children who underwent a RS procedure for HSCR at our centre from 2015 to 2025. The primary aim of the study was to describe clinical outcomes following implementation of robotic surgery for HSCR at our institution. To provide clinical context, outcomes were compared with those of patients who underwent laparoscopic surgery during the same period.

## Material and methods

All children (aged 0–18 years) diagnosed with HSCR who consecutively underwent robotic-assisted surgical procedure at our Institution from October 2015 to July 2025 were included and retrospectively analysed.

The recorded data included patient demographics, clinical details and diagnostic workup. As part of the preoperative evaluation, all patients underwent rectal biopsies, contrast enema, and echocardiography. Data about surgical details, intraoperative complications and need for conversion were collected. If present, intestinal stoma closure was performed either during the same procedure or as a staged operation according to institutional clinical practice, taking into account patient clinical condition, bowel status, and surgeon judgment.

Intraoperative times were recorded:

- Total operative time – from skin incision to skin closure,
- Console operative time – duration of the surgeon's active operation at the robotic console.

Postoperative data included: drain placement, postoperative complications graded more than 3a according to Clavien-Madadi classification (5), postoperative outcomes (constipation, soiling, incontinence), and length of follow up.

Follow-up usually included a rectal examination under general anesthesia 1 month after surgery, and periodical clinical examination at 3 and 6 months postoperatively, and annual assessments thereafter.

## Surgery

The operating surgeons were paediatric surgeons with substantial experience in HSCR surgery, in both open and conventional MIS.

**Figure 1:**
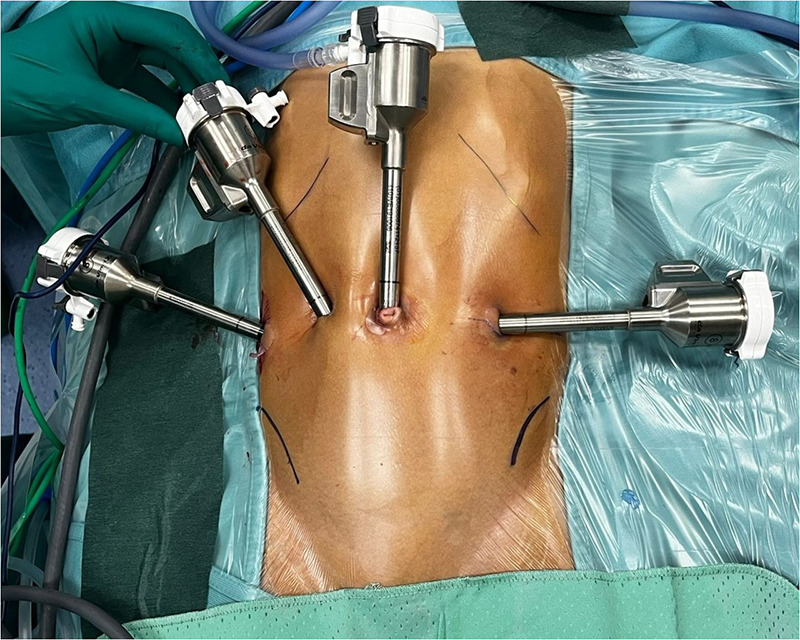
Robotic trocars placement (along the transverse umbilical line) for robot-assisted laparoscopic surgery for Hirschsprung disease.

All procedures were performed using the da Vinci Xi® Surgical System (Intuitive Surgical Inc., Sunnyvale, CA, USA). Console operative time was performed by one surgeon (GM), already expert in gastrointestinal RS, while perineal phase was performed by a younger surgeon, already experienced in HSCR's surgery (MGF, VR). The patient was placed in a supine position with the perineum included in the operative field. Four 8-mm robotic trocars were placed along the transverse umbilical line or, in smaller children, along a supraumbilical transverse line. (Figure 1) Pneumoperitoneum was set up and maintained at a pressure of 10-12 mmHg. A seromuscular biopsy was performed approximately 5 cm above the transition zone and sent for intraoperative frozen-section pathological examination to assess the presence of ganglion cells. Patients affected by rectosigmoid HSCR underwent ERPT according to the Soave-Georgeson technique (SG) at the beginning of the study period, whereas a Swenson-like ERPT with minimal endorectal dissection (SW) was adopted in subsequent cases. For long-segment HSCR, a SW combined with a Deloyers’ maneuver (DL) was performed, while ERPT with ileoanal anastomosis was used in one case of redo surgery for total colonic aganglionosis (TCA). One patient underwent robotic cuff excision following a previous Soave ERPT.

## Statistical analysis

Descriptive data are presented as means with standard deviation or medians for continuous variables, and as absolute numbers with corresponding percentages for categorical variables. A comparative analysis was performed with patients who underwent laparoscopic surgery for HSCR. Comparisons between categorical variables were made using the chi-squared test. Statistical significance was defined as *p* ≤ 0.05.

## Results

A total of 53 patients affected by HSCR were treated at our Institution from October 2015 to July 2025, twenty (38%) of them underwent a robotic-assisted surgical procedure and were prospectively included in the series and retrospectively analysed.

The median age at the time of surgery was 1.3 years (IQ range 0.6-4.8), with a mean weight of 16 kg. The youngest patient was 4 months old and weighed 6 kg.

- Seven-teen patients were affected by rectosigmoid HSCR (85%), of them twelve patients underwent a SG. Four of them underwent a SW and one a cuff excision after a Soave ERPT.
- Two patients were affected by long-segment HSCR (10%); two of them underwent a SW associated with DL.
- One patient with a misdiagnosed TCA (5%) underwent a robotic redo ERPT with ileo-anal anastomosis following a previous Soave ERPT.

**Figure 2:**
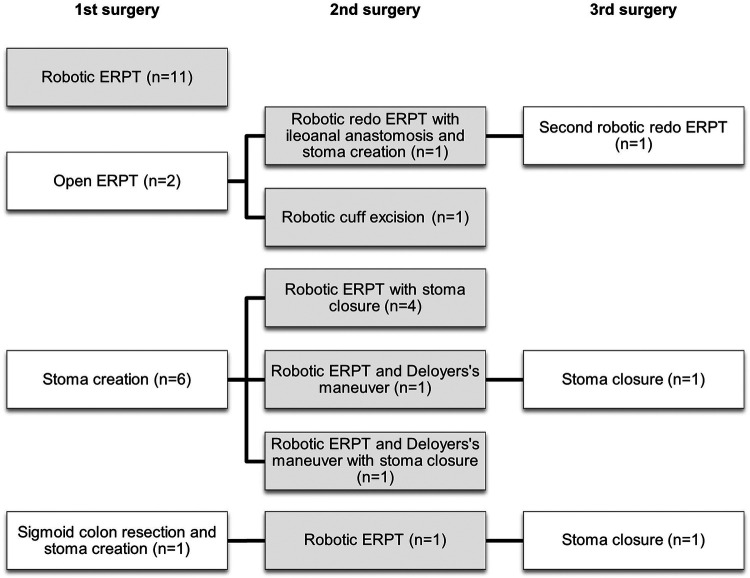
Surgical history of the 20 patients. Grey boxes = robotic procedure analysed in the study. ERPT, endorectal-pull through.

Operative times analysis showed a median total operative time of 253 minutes (IQ range 188-402), and a median console operative time of 50 minutes (IQ range 40-95). No intraoperative complications occurred. Demographic, and operative data are showed in Tables 1, 2. The surgical history of all operated children, including the index RS procedure and any previous or subsequent interventions is shown in Figure 2.

**Table 1:** Demographic, and preoperative data.

Demographic and clinical variables before surgery	Patients (*n* = 20)
Age at surgery(*years*),*median (IQR)*	1.3 (0.6-4.8)
Gender	
Male, *N* (%)	14 (70%)
Female, *N* (%)	6 (30%)
Weight *(kg), mean ± SD*	16 ± 15
Failure to pass meconium before 24 hours*, N (%)*	9 (45%)
Late diagnosis, &gt; 1 year of age, *N* (%)	8 (40%)
Disease extension	
Rectosigmoid HSCR, *N* (%)	17 (85%)
Long-segment HSCR, *N* (%)	2 (10%)
TCA, *N* (%)	1 (5%)
Enema, *N* (%)	20 (100%)
Type of diagnostic biopsies	
Suction, *N* (%)	19 (95%)
Open, *N* (%)	1 (5%)
HAEC before surgery, *N* (%)	6 (30%)
Cardiopathies, *N* (%)	1 (5%)
Urogenital anomalies, *N* (%)	0
Ipoacusia, *N* (%)	0
Sindrome, *N* (%)	0
Genetic anomaly, *N* (%)	0

IQR, interquartile range; HSCR, Hirschsprung disease; HAEC, Hirschsprung-associated enterocolitis, TCA, total colonic aganglionosis.

**Table 2:** Intraoperative variables and surgical procedures.

Intraoperative data	Patients (*n* = 20)
First surgery, *N* (%)	11 (55%)
First ERPT, *N* (%)	18 (90%)
Redo ERPT, *N* (%)	1 (5%)
Type of robotic procedure	
SG, *N* (%)	12 (60%)
SW, *N* (%)	4 (20%)
SW with DL, *N* (%)	2 (10%)
ERPT and ileo-anal anastomosis, *N* (%)	1 (5%)
Cuff excision, *N* (%)	1 (5%)
Console operative time*(minutes), median (IQR)*	50 (40-95) [*n* = 18]
Total operative time*(minutes), median (IQR)*	253 (188-402) [*n* = 13]
Conversion, *N* (%)	1 (5%)
Intraoperative complications ≥ 3a, *N* (%)	0
Drain placement, *N* (%)	2 (10%)
Intestinal stoma, performed before or at the same time of ERPT, *N* (%)	8 (40%)
Type of stoma	
Ileostomy, *N*	6
Colostomy, *N*	2
Stoma closure during ERPT, *N* (%)	5 (25%)
Stoma closure after ERPT, *N* (%)	2 (10%)
Stoma still present at last follow-up, *N* (%)	1 (5%)

ERPT, endorectal-pull through; SG, Soave-Georgeson ERPT; SW, Swenson-like ERPT with minimal endorectal dissection; DL, Deloyers’ maneuver; IQR, interquartile range.

Postoperative complications of Clavien-Madadi grade ≥ 3a occurred in 6 patients. Five patients developed a mild anastomotic stenosis, which were managed with Hegar dilations. One patient had a redo ileoanal anastomosis due to dehiscence.

The median length of follow-up was 11 months (IQ range 5–12). Three patients were lost to long-term follow-up. Among the sixteen patients with available data and no stoma, two patients complained mild constipation with soiling episodes; and two had experienced episodes of HAEC.

Surgical success rate was 95%.

Postoperative data are showed in Table 3.

**Table 3:** Postoperative data, and clinical variables at last follow-Up.

Postoperative data	Patients	N
Postoperative complications		
Overall, *N* (%)	6 (30%)	[*n* = 20]
Anastomotic stenosis, *N* (%)	5 (25%) → Hegar dilations	[*n* = 20]
Cuff stenosis, *N* (%)	0	[*n* = 20]
Dehiscence, *N* (%)	1 (5%) →Redo ileoanal anastomosis	[*n* = 20]
Surgery failure (Need for redo surgery), *N* (%)	1 (5%)	[*n* = 20]
Postoperative issues at last follow-up, *N* (%)^a^		
Severe constipation, *N* (%)	0	[*n* = 17]
Mild constipation with soiling, *N* (%)	2 (12%)	[*n* = 16]
Incontinence, *N* (%)	0	[*n* = 17]
HAEC after surgery, *N* (%)	2 (12%)	[*n* = 17]
Length of follow-up *(months),* *median (IQR)*	11 (5-12)	

a long term follow-up data available for 17 patients; HAEC, Hirschsprung-associated enterocolitis; IQR, interquartile range.

## Comparison with laparoscopic surgery

Comparison with 24 patients who underwent Soave–Georgesson ERPT during the same study period revealed no statistically significant differences in rates of anastomotic stenosis, anastomotic dehiscence, HAEC, or mild constipation with soiling. These results are summarized in Table 4.

**Table 4:** Comparison of operative outcomes between patients who underwent robotic surgery for hSCR and those who underwent laparoscopic surgery.

Outcomes	Robotic Group [*n* = 20]	Laparoscopic Group [*n* = 24]	*p-value*
Postoperative complications			
Anastomotic stenosis, *N* (%)	5 (25%)	2 (8%)	0.13
Dehiscence, *N* (%)	1 (5%)	1 (4%)	0.19
Postoperative issues at last follow-up			
HAEC after surgery, *N* (%)	2 (10%)	3 (12%)	0.79
Severe constipation, *N* (%)	0	0	
Mild constipation with soiling, *N* (%)	2 (10%)	3 (12%)	0.79

HAEC, Hirschsprung-associated enterocolitis.

## Discussion

The present study describes the real-world implementation of RS for HSRC in a pediatric surgical center over a ten-year period, focusing on feasibility, surgical application, and early clinical outcomes rather than on a formal comparison between surgical approaches.

Since its introduction, RS had expanded across nearly all areas of pediatric surgery; however, its adoption had progressed more slowly than in adult surgery. Several subfields and indications are still under evaluation—particularly in neonates, due to instrument size limitations, and in certain subspecialties, where evidence is limited by the absence of large case series (6, 7).

The high costs of RS have limited and delayed its widespread adoption, and the cost difference between RS and laparoscopy for HSCR is statistically significant (8). However, as reported for other surgical indications, once the learning curve has been overcome, increased robotic experience may lead to shorter hospital stays and fewer long-term complications, potentially offsetting the initial investment in equipment. While the robotic approach is well established and has proven advantages over conventional minimally invasive and open surgery for certain pediatric urology procedures, having been introduced in the early 2000s, its adoption in pediatric colorectal surgery occurred roughly ten years later (2). Large series or comparative studies defining clear indications remain limited, although the safety and feasibility of this approach have been demonstrated.

A systematic review reported that robotic colorectal surgery is a safe and effective approach in children, particularly for complex conditions such as HSCR disease, inflammatory bowel disease, and anorectal malformations (9).

The first reported use of RS for HSCR was in 2011 by Hebra et al. Their series included 12 infants who underwent a Swenson ERPT, with a mean age of 16 weeks and a mean weight of 5.5 kg. The average operative time reported was 230 minutes. Six patients needed dilations for minor rectal strictures (2).

Since then, a number of retrospective studies have been published reporting various applications of RS for HSCR, including Swenson and Soave ERPT, Duhamel pull-through, Deloyers maneuver, innervation mapping, redo surgery, excision of a redundant rectal pouch, and radical proctocolectomy (10–15).

Three recent systematic review and meta-analysis of Li et al., Langeron et al., and Durazo et al. compared RS and laparoscopy in the treatment of HSCR (4, 16, 17). Four studies are included in all three reviews.

The systematic review and meta-analysis of Li et al. included 6 studies (3, 8, 18–21) involving 789 children (352 in RS group) and showed significantly less intraoperative blood loss in the RS group but longer operative time. No significant group differences were found in postoperative HAEC, anastomotic complications, soiling, and wound infection. Hospital stay was significantly shorter in the RS group (16). Durazo et al. included four of the six studies analysed by Li et al. and reached similar conclusions (17).

The systematic review of Langeron et al. assessed not only the comparison between RS and laparoscopy for HSCR, including five studies (3, 8, 12, 18, 20), but also categorized patients into three groups: infants, older children, and redo surgeries, in order to evaluate indications and limitations of RS in HSCR. The authors concluded that RS appears feasible and safe in infants, although it does not show superiority over laparoscopy in this age group. In older children and redo cases, however, RS provides advantages due to more challenging rectal dissection in a larger pelvis and/or the presence of scar tissue (4). Rectal surgery represents a well-established indication for minimally invasive techniques, given the confined operative space within the pelvis, particularly in pediatric patients. Moreover, these procedures are often prolonged, and the robotic platform may provide technical advantages by reducing surgeon fatigue and facilitating complex maneuvers, such as distal rectal dissection in the Swenson plane, thereby potentially allowing for a more nerve-sparing approach. In the comparative analysis between RS and laparoscopy, they reported significantly lower intraoperative blood loss, longer postoperative fasting time, and higher hospitalization costs. For complications, their meta-analysis showed no differences between the two groups, consistent with the findings of Li et al. (16).

The authors of reported reviews concluded that available data do not support clear and strong clinical recommendations, especially for lack of data about the benefits of RS on long term functional outcomes.

Our series reflects the real-world integration of RS into pediatric colorectal practice rather than a highly selected feasibility cohort. We report our series of 20 children who underwent RS for HSCR over a period of ten years (2015–2025) in a single center, aiming to contribute to the available data on these new frontiers in pediatric surgery. In our study population, demographic and preoperative characteristics were consistent with those reported in the literature for patients affected by HSCR, except for a higher proportion of older children (40%). This finding may be explained by a selection bias, as patients selected for RS at the beginning of the LC tend to be older due to instrument size limitations and technical ease in this age group. The distribution of disease extension in our cases perfectly reflects that of the broader HSCR population: 85% rectosigmoid HSCR, 10% long-segment HSCR, and 5% TCA (1, 22). This fortunate resemblance makes the sample especially valuable for demonstrating how RS and potential outcomes can be applied to the HSCR population.

Surgical history in HSCR patients can be complex. Figure 2 summarizes the surgical history of our patients. In our sample, 11 patients had undergone a single surgery, while nine (45%) had more than one procedure. This highlights the applicability of RS in previously operated patients, as reported in the literature, owing to its advantages: a more ergonomic platform suitable for lengthy procedures, high-resolution three-dimensional vision, tremor filtration, and instruments with wrist-like articulation, which are particularly useful in confined spaces such as the pediatric pelvis. Nonetheless*,* the principal limitation of the robotic approach is the absence of force feedback in the da Vinci system, which requires careful modulation of tissue traction to minimize the risk of inadvertent injury (13).

Regarding surgical procedures, we performed 19 ERPTs. At the beginning of our experience, we translated our usual technique to RS, which was the SG. With increasing experience, we found that keeping a safe Swenson plane in the abdominal phase is considerably easier with RS than with laparoscopy, with a lower risk of nerve and vascular injury. Consequently, the technique was gradually modified to a SW, which is now our standard robotic ERPT approach. This modification is supported by literature on RS and aligns with current ERNICA guidelines, which recommend avoiding long seromuscular cuffs (1, 19).

Among the seven patients who underwent a SW, we also treated three children with long-segment HSCR combining a DL. In these cases, trocars were placed in the upper abdomen to allow 180 degrees rotation of the right colon before proceeding with the pull-through, and indocyanine green was used for assessing vascularization of the remaining colon. Patients included in the series had an uneventful postoperative course and are progressing well. The first case of this robotic maneuver in a pediatric patient is included in this series and was already published (15).

The feasibility of ERPT with ileoanal anastomosis had been already reported in literature for total colonic aganglionosis (11), and was successfully applied in one redo patient from this series with residual aganglionosis following a previous Soave ERPT.

On total operative time, Langeron et al. (4) reported a median of 207 minutes in their meta-analysis, which is consistent with our median operative time of 253 minutes. Console operative time was not reported in the cited systematic reviews. In our series, the median console time was 50 minutes (IQR 40–95), while the mean console time was 69 ± 45.8 minutes. Compared to Zhang M. et al.'s cohort of 156 robotic ERPT patients (mean console time 58.01 ± 7.71 minutes), our mean console time (69 ± 46 minutes) was similar. However, interpretation should consider differences in sample size and variability.

The incidence of postoperative complications in the literature varies, likely due to heterogeneity in follow-up duration and differences in patient populations. Complication, leading to reintervention, in this series (5%) overlaps those reported in literature, ranging from 0% to 10% (3, 14, 16, 20, 23), and these rates are not higher than those observed after laparoscopic procedures, according to the systematic reviews (4, 16, 17).

HAEC has also been reported following RS for HSCR, with an incidence ranging from 5% to 20% (3, 11, 14, 16, 24). In our cohort, two patients experienced postoperative HAEC, and this is consistent with literature.

Zhang M. et al. (14) reported a large, homogeneous cohort of patients all undergoing ERPT and provided detailed bowel-function outcomes at medium-term follow-up. Using a scoring system, they reported good bowel function in 75% of patients and moderate function in 16%. Specifically, they found constipation in 7% and soiling in 16% of their cohort. In comparison, we observed mild constipation with episodes of soiling in two patients (12%) at the last follow-up, although Zhang et al. reported outcomes after a longer follow-up period.

A comparative analysis with postoperative outcomes observed in patients undergoing laparoscopic Soave-Georgesson ERPT at our centre during the same period showed comparable results.

## Limitations of the study

The present study has several limitations. First, the relatively small sample size and the single-center design limits the generalizability of the findings. Second, the retrospective nature of the analysis may introduce selection bias, as treatment allocation and surgical strategy were based on clinical judgment rather than predefined criteria. In addition, the heterogeneity of patients in terms of age, disease extension, and surgical indication reflects real-world clinical practice but limits the possibility of standardized comparisons. Moreover, because these cases represent initial experience of a pediatric surgeon already experienced in HSCR surgery but newly adopting this technique, the outcomes may be influenced by the introduction of a new surgical approach. Finally, the comparison with the patients operated through laparoscopic approach has a selection bias, due to that fact the patients of laparoscopic group underwent all to the same procedure and tend to be smaller.

## Conclusions

According to both the literature and our experience, RS is a feasible possibility for the management of HSCR and represents a promising surgical approach. At our centre, RS has yielded outcomes comparable to those of laparoscopy, while the literature suggests that its potential advantages may be emerging in more complex cases. However, long-term outcomes do not yet appear to differ between the two techniques. We believe that RS is still in its LC phase, and further studies involving surgeons already experienced with this method will be necessary to accurately assess its long-term results.

## Data Availability

The raw data supporting the conclusions of this article will be made available by the authors, without undue reservation.
